# An Analysis of Monitoring Solutions for CAR T Cell Production

**DOI:** 10.1049/htl2.70012

**Published:** 2025-05-13

**Authors:** Arber Shoshi, Yuchen Xia, Andrea Fieschi, Yannick Baumgarten, Andrea Gaißler, Thomas Ackermann, Peter Reimann, Bernhard Mitschang, Michael Weyrich, Thomas Bauernhansl, Robert Miehe

**Affiliations:** ^1^ Graduate School of Excellence advanced Manufacturing Engineering (GSaME) University of Stuttgart Stuttgart Germany; ^2^ Fraunhofer Institute for Manufacturing Engineering and Automation (IPA) Stuttgart Germany; ^3^ Institute of Industrial Manufacturing and Management (IFF) University of Stuttgart Stuttgart Germany; ^4^ Institute of Industrial Automation and Software Engineering (IAS) University of Stuttgart Stuttgart Germany; ^5^ Institute for Parallel and Distributed Systems (IPVS) University of Stuttgart Stuttgart Germany; ^6^ Mercedes Benz Stuttgart Germany

**Keywords:** advanced therapy medicinal product (ATMP), CAR T cell therapy, monitoring, on‐line measurement

## Abstract

The chimeric antigen receptor T cell (CAR T) therapy has shown remarkable results in treating certain cancers. It involves genetically modifying a patient's T cells to recognize and attack cancer cells. Despite its potential, CAR T cell therapy is complex and costly and requires the integration of multiple technologies and specialized equipment. Further research is needed to achieve the maximum potential of CAR T cell therapies and to develop effective and efficient methods for their production. This paper presents an overview of current measurement methods used in the key steps of the production of CAR T cells. The study aims to assess the state of the art in monitoring solutions and identify their potential for online monitoring. The results of this paper contribute to the understanding of measurement methods in CAR T cell manufacturing and identify areas where on‐line monitoring can be improved. Thus, this research facilitates progress toward the development of effective monitoring of CAR T cell therapies.

## Graphical abstract

The image schematically shows the convergence of the three levels ‐ the physical level, the monitoring level and the digital level ‐ with the aim of achieving the vision of Pharma 4.0, a fully networked, data‐driven and automated production environment. In this work, we focus on the physical level and the monitoring level. We explore how automation and monitoring can be integrated in CAR‐T cell production to achieve greater efficiency, quality assurance, and improved patient outcomes.

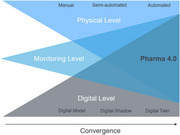

## 1 Introduction

Despite the significant clinical success of ATMPs, their widespread adoption remains limited. In 2021, only 15 ATMPs were approved in the European Union, while five others were withdrawn due to economic constraints [1]. Among ATMPs, CAR T therapies have shown exceptional outcomes in treating hematologic malignancies such as B‐cell acute lymphoblastic leukemia and B‐cell lymphoma. However, their application remains constrained by high production costs, complex manufacturing processes, and limited scalability. CAR T cells are produced through the ex vivo genetic modification of T lymphocytes to express tumor‐targeting chimeric receptors. In autologous approaches, T cells are derived from the patient and re‐infused after engineering. While this personalized strategy offers clinical benefits, it presents manufacturing challenges, including variability in starting material, stringent safety requirements, and a multi‐step production process involving activation, transduction, expansion, and formulation [2, 3, 4, 5]. The high cost of therapy—up to €475,000 per treatment plus hospitalization expenses—can be attributed to labor‐intensive manual steps, specialized infrastructure, and the complexity of individualized logistics [6, 7, 8]. As demand for CAR T‐cell therapies grows, current manufacturing capacities may be insufficient to meet clinical needs [2]. Achieving scalable and cost‐effective production will require automation, modularity, and seamless integration of sub‐processes [8].

Recent efforts in biopharmaceutical manufacturing point toward digitalized production systems, aligning with the principles of Pharma 4.0 [9]. These systems integrate automated control, machine‐to‐machine communication, and advanced monitoring to improve reproducibility and reduce batch failure. For CAR T‐cell therapy, efficient process monitoring plays a key role in optimizing production, identifying inefficiencies, and ensuring product safety and quality. Continuous monitoring and analysis allow the optimization of the production process and the improvement of the design of production systems by identifying bottlenecks and inefficiencies in the production process [10, 11]. Research into real‐time and on‐line monitoring tools is therefore critical.

This research paper highlights the importance of effective monitoring of key parameters in CAR‐T cell production and evaluates different monitoring solutions that can be implemented to improve the quality and reliability of the manufacturing process of CAR‐T cells. The analysis covers the intricacies involved in the automated and digitized production of CAR‐T cells, outlining different production methods and their critical procedural elements. Additionally, this study investigates monitoring methods, with a particular emphasis on areas where current digital monitoring techniques are still underdeveloped. The paper presents research findings and insights into the promising use of monitoring methods for CAR‐T cell production.

## 2 Methodology

For the purpose of analysing monitoring solutions in CAR‐T cell production [12, 13], was conducted using PRISMA analysis [14] as the research methodology. The choice of the approach was based on its comprehensive and open data collection and evaluation techniques, facilitating maximum information capturing. This paper uses subjective perception to describe and define concepts, abstraction based on individual cases, and the development of alternative actions to identify future realities. Through examining and analysing specific aspects of the world, various options for future goals can be developed. Based on the literature review and data analysis results, an overview of the parameters to be monitored in CAR T cell production and possible monitoring methods were developed.

Additionally, to enable a decentralized and modular production process, the main steps of the previously non‐standardized CAR T cell production process were identified, and a coherent theoretical data model of a future decentralized production system was developed. The research process used in this study is similar to approaches documented in the literature [15, 16]. The research paper aims to contribute to the knowledge base on on‐line monitoring solutions by a morphological box‐like measurement approach for ATMPs, especially the CAR T therapy, and provide insights for future research and development.

## 3 Enabling Technologies for Digitized Manufacturing of CAR T Cells

In technological advancements across diverse production landscapes, three critical components emerge: automation, real‐time monitoring, and digitalization. This trend is also evident in biomanufacturing [17, 18, 19]. These elements’ synergy can potentially revolutionize biomanufacturing methods, elevating efficiency, safety, and more [18]. While these components are versatile across production areas, this discussion will focus on their application in CAR T cell production.

Automation plays a pivotal role by minimizing human involvement in various processes. This not only optimizes workflow efficiency but also reduces potential errors [20]. Automation ensures uniformity in the production process, regardless of its intricacy. This consistency is especially vital in biomanufacturing, where minor procedural deviations can result in significantly varied outcomes [21]. Moreover, automated systems provide scalability, addressing growing demands without sacrificing quality [20].

Monitoring, even independent of automation, is integral to quality assurance, identifying standard deviations, and ensuring safety. Its significance lies in maintaining consistency, reliability, and circumventing possible pitfalls, such as process variations, equipment malfunctions [22], and human errors [23]. When integrated with automation, monitoring diligently oversees the flawless execution of automated tasks. Its scope extends beyond anomaly spotting to preemptively mitigating potential deviations [24]. In CAR T cell production, both precision and traceability are crucial. Implementing a monitoring system aids in identifying deviations, tracking the product's location, and assessing its status, facilitating a more agile and automated production. This adherence ensures compliance with regulatory standards [24, 25].

Digitalization complements the monitoring framework. It is not about mere data aggregation but data enhancement, capturing intricate details like temperature variances during cell modifications and tracking production timelines. This data serves as an invaluable analytical asset [26]. Leveraging sophisticated algorithms, insights from this data can refine subsequent production methodologies [27].

**FIGURE 1:**
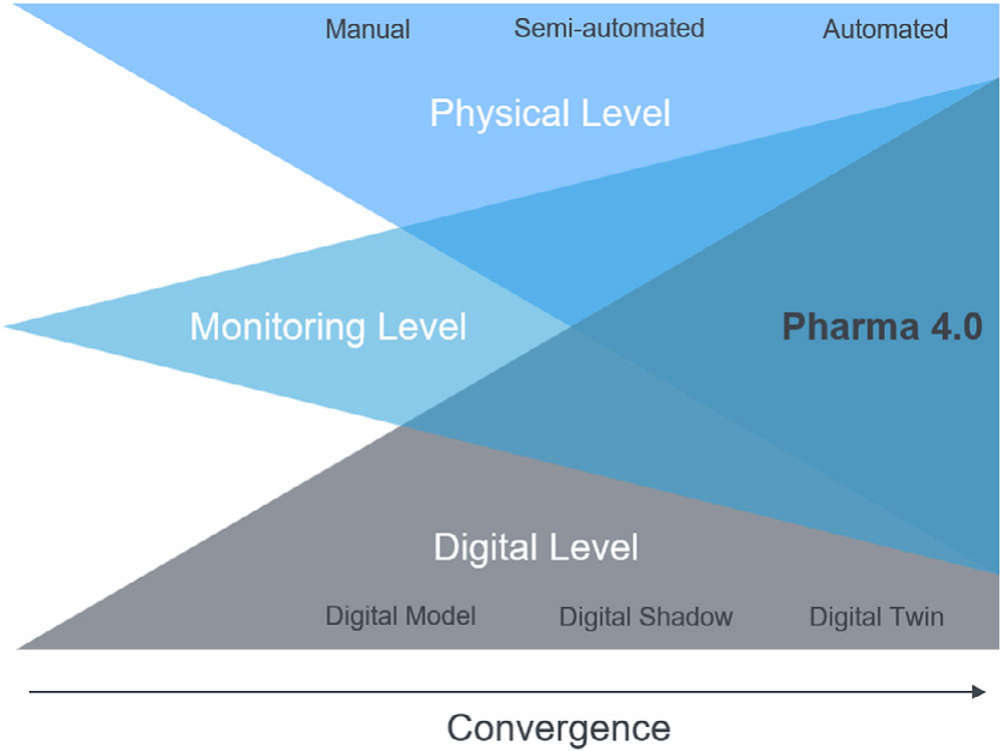
The increase and convergence of the three entities automation, digitalization, and monitoring leading to the concept of Pharma 4.0.

The pharmaceutical industry has undergone consistent technological advances, leading to the current era of “Pharma 4.0”. As shown in Figure 1, this paradigm integrates the three mentioned entities automation, monitoring, and digitalization to address challenges in the biopharmaceutical supply chain. Interconnected systems are enabling this transformation characterized by continuous data communication. Such systems integrate cyber‐physical components, where data science tools play a crucial role [9, 28]. The aim of the technology is seen to pivot in the idea of biointelligent manufacturing. Biointelligent systems are (commonly) defined as the convergence of technical, biological, and informational elements [29]. This biological transformation of technology extracts knowledge from nature and applies it to technology, yielding systems such as decentralized production cells capable of producing personalized, biobased products. For example, on‐site bioreactors can create custom medications in pharmacies [15], like CAR T cell therapy. This approach aims at a more streamlined CAR T cell production, holding promise for enhanced patient outcomes globally. This study primarily delves into the automation and monitoring facets, laying the groundwork for an evolved CAR T cell production process.

## 4 Major steps in the CAR T Cell Production

**FIGURE 2:**
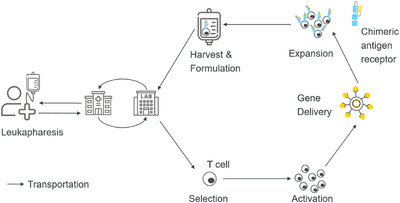
The procedure for generating allogeneic CAR T cells from the hospital into the laboratory and back to the hospital. There, the cells are transformed into CAR T cells and subsequently returned to the hospital for transfusion to the patient. Image adapted from [2].

The production of CAR‐T cells is an unstandardized process that typically involves five primary stages: selection, activation, gene transfer, expansion, and formulation. Figure 2 depicts the main steps of the CAR T production process.

In the first step, T cells are isolated from leukapheresis products, for example, using magnetic bead isolation. Leukapheresis products refer to blood components separated through a medical procedure, usually for therapeutic purposes. Then, in the next step T cells are activated using, for example, beads or a combination of monoclonal antibodies and IL‐2 [30]. IL‐2, or interleukin‐2, is a cytokine protein that plays a crucial role in immune system regulation.

The third step is gene delivery, which aims to safely and efficiently introduce the CAR gene mainly using viral vectors [30]. The fourth step is the expansion of CAR T cells. This step is the most time‐consuming. The cells are cultivated for up to 14 days in the presence of growth factors and other nutrients to achieve the required cell number for reinfusion into patients. The expansion of transduced or transfected CAR T cells is a crucial step in which the number of cells is increased by changing the culture volume. This is possible with several methods, such as using multiple tissue culture plates or flasks, a larger number of vessels, or static culture bags. The rocking motion (RM) bioreactor is a further method that allows cultures in smaller volumes, resulting in a final product with more CD4+ T cells [16, 17].

The final step, harvesting of cells and formulation, must be sterile and free of contaminants to ensure patient safety. This requires ensuring that the final product meets regulatory requirements for safety, purity, and potency [30, 31]. Formulation is typically carried out in a controlled environment within a cleanroom or sterile facility. In this process, the cells are washed to remove any residual culture media and reagents that could cause adverse reactions in the patient. The cells are then resuspended in a solution and frozen, which stabilizes the cells during storage (until sterility testing is performed [32]) and transport to the hospital, where they are infused into the patient.

While this manuscript primarily focuses on autologous CAR T‐cell therapies, it is important to acknowledge the distinct safety challenges presented by allogeneic approaches. Autologous therapies utilize a patient's own T cells, minimizing the risk of immunogenic reactions; however, they entail complex individualized manufacturing processes. In contrast, allogeneic therapies use donor‐derived cells, offering potential benefits in scalability and cost‐efficiency but posing significant risks such as graft‐versus‐host disease (GvHD), immunogenicity, and increased infection susceptibility. These challenges necessitate careful monitoring strategies tailored to the specific risks associated with each approach, reinforcing the need for advanced, integrated, real‐time monitoring solutions to ensure safety and efficacy in CAR T cell manufacturing.

### 4.1 Manufacturing Principles

Autologous CAR T cell production is small‐scale batch production, and the outcome of the production is determined in each step. For example, by the choice of activation or gene delivery methods [30]. Additionally, the manufacturing of CAR T cells must be performed under good manufacturing practice (GMP) conditions and is primarily performed in a manual or semi‐automated manner [2]. In this chapter, we would like to introduce manual and semi‐automated production principles and discuss the possibilities of a fully‐automated cell and gene therapy production of the future.

#### 4.1.1 Manual Production of CAR T Cells

As discussed above, CAR T cells are produced in many steps and are therefore very labor‐intensive. Culturing T cells in T‐Flasks or other vessels (for example, the G‐Rex System) requires frequent medium exchange. This open handling step needs well‐trained operators and is usually performed in cleanrooms to avoid contamination of the final product [30]. While culturing of T cells in small flasks might be suitable for autologous single‐batch CAR T cell production, scaling up and transferring the process to allogeneic CAR T production is not feasible due to the variety of open handling steps [30].

#### 4.1.2 (Semi‐) Automated CAR T Cell Production

At the moment, six CAR T cell products are approved in Europe [33], and there are currently more than 400 clinical gene therapy trials ongoing worldwide [34]. This number of clinical trials and potential therapies highlights the need for efficient production of cell‐ and gene therapies.

To reduce time, costs, and the number of open handling steps, static culture bags or rocking motion bioreactors can be connected via tubing in a sterile manner [30]. Semi‐automated CAR T production solutions allow the connection of existing equipment [35] to decrease open handling steps, thereby increasing standardization within an organization [30]. However, the choice of the culture vessel might impact the final product: it was shown that rocking motion bioreactors might influence the CD4:CD8 CAR T cell ratio during expansion [36].

The G‐Rex System includes an automated media collection system that allows for transferring fluids from one vessel to another via sterile welding of tubes [37]. There are various systems on the market that automize single steps of the manufacturing process, but there are also new production concepts in the pipeline [37]. One example is an isolator‐based approach that reduces time and costs and has the potential to be fully automated [38].

There are also all‐in‐one devices on the market that cover all process steps at once using the CliniMACS Prodigy® or the ekkoTM system. T cell isolation, gene editing, and cultivation can be performed in one device [2, 37, 39]. This reduces costs and contamination risks [2, 39]. However, these devices have only basic QC integrated (for example, temperature or pH value) and lack cellular QC parameters like CAR expression or cell viability [2]. To assess these parameters, samples are manually taken and analysed off‐line [2]. The Lonza Cocoon® Platform is a similar automated cell therapy manufacturing platform, which is additionally easily scalable with a small footprint [37].

#### 4.1.3 Efficient CAR T Cell Production of the Future

Manual or semi‐automated production processes cause high costs and product variability. Using production technologies described in the previous section, sterility testing and characterization of the final CAR T product are not performed on‐ or in‐line but after the cells have been produced [32].

A fully automated and efficient CAR T cell production requires new hardware that allows on‐or in‐line monitoring of the process and has standardized interfaces to connect the different devices and enable digitalization of the process [2, 35]. Label‐free on‐line detection of cell number, viability, and even different cell states in combination with several kinds of bioreactors [40] can be performed in CAR T production, but literature on GMP production processes using these devices remains scarce. For future applications, for example, in the allogeneic CAR T production, the manufacturing process must be scalable [30]. CAR T cell products must be sterile, free of debris and unlabeled. Therefore, new hardware must be fully automated with standardized interfaces and enable label‐free control of the cell at any time and at any step of the production process. Ideally, the collection of this data allows the creation of digital twins, enabling an automatic and intelligent process control adapted to the patients’ cells. Lately, modular and scalable platforms have been developed, for example, by the company's cellular origins. This platform can be adjusted to the customers’ needs and operated manually or by an autonomous robot [41].

A concept of a fully automated, modular cell, and (cell‐based) gene therapy production in a so‐called pharming matrix has previously been described [35]. Here, each process step is realized in a module, and the cells, the required liquids, and vessels are enclosed in cassettes. Each cassette is compatible with each processing module [35]. Thereby, autologous CAR T cell and allogeneic products can be produced through scaling up via the use of multiple cassettes per production line. Furthermore, for other cellular ATMPs, the sequence of the production step might be different, and the modules can be utilized accordingly [35]. This system is fully digitalized through the use of real‐time in‐line sensors. The availability of the needed sensor technologies is discussed in the following sections.

### 4.2 Monitoring Methods for the CAR T Cell Production

**FIGURE 3:**
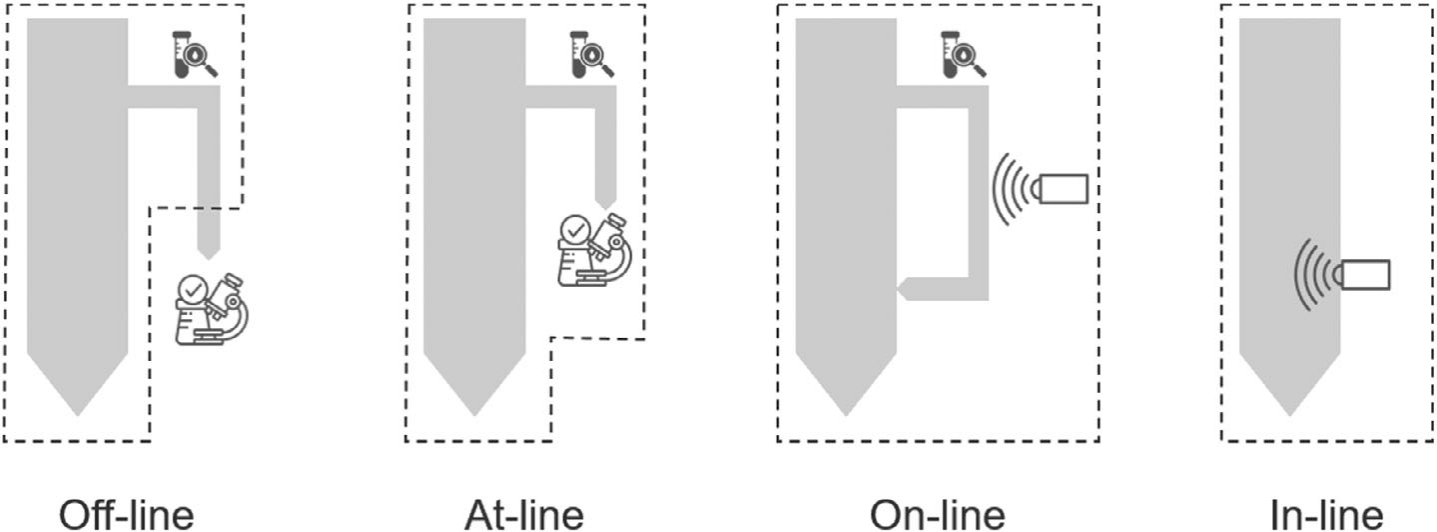
Various methods of analysis. Off‐line measurements require collecting samples and conducting laboratory analysis. At‐line measurements bring the laboratory to the process site but still require sample collection. On the other hand, on‐line and in‐line methods involve direct analysis. In on‐line measurements, slipstream techniques like bypasses are needed, while in‐line measurement is directly integrated. Illustration based on [49, 50].

The biotechnology industry, especially the biopharmaceutical industry, is still far from being fully automated. Within the industry many parameters of the production processes are measured with great manual effort [42, 43]. Figure 3 illustrates the four basic types of analytical methods currently used in biopharmaceutical production. Off‐line and at‐line measurements involve the analysis of samples outside or near the production facility [44, 45]. Unlike on‐line and in‐line measurements, the collected sample is not reintroduced into the production process and cannot be used later for the patient's therapy. In‐line and on‐line measurements allow real‐time analysis of process parameters. In on‐line measurement, a sample is automatically examined in a bypass and then returned to the production process, provided it has not been contaminated by additives or foreign substances [46, 47, 48]. In‐line measurement is the most desirable, performed directly on the production line with minimal effort [44]. To achieve proper on‐line measurement, the measuring instruments must be integrated into the production process.

A literature review was conducted to identify prevalent methods utilized for monitoring CAR T cell production processes. Table 1 presents the identified methods along with the typical data formats for each measurement. The file format, which includes standardized and proprietary formats to store information, is a crucial aspect of providing a comprehensive overview of the diverse data formats used to monitor CAR T cell production. The most commonly used data formats for storing CAR T cell production monitoring data are CSV and TXT files, which express the data in tabular form. These and other file formats are abbreviated and described in lowercase letters in the table's footer. In addition, the table indicates whether each measurement method is off‐line, at‐line, on‐line, or in‐line. This information is significant, as it highlights the points in the production process where real‐time measurements are not expected to be feasible. Furthermore, the table indicates if the measurement method has real‐time capability. Real‐time capability refers to the ability of a measurement method to provide immediate and continuous data, allowing for instantaneous monitoring and control during a production process [51]. This feature is particularly valuable in ensuring timely adjustments and maintaining the quality and efficiency of the production workflow. The current order in Table 1 from off‐line to in‐line monitoring methods reflects the typical progression of technological maturity in the field and illustrates the evolution from traditional approaches toward future‐ready, integrated monitoring systems.

**TABLE 1:** Methods used for measuring key parameters in the CAR T cell production. The monitoring readiness (MR) is categories in three types. The categories: (I) (this measurement method has been used I CAR T cell production); (II) (this measurement has been used in production of other ATMPs); and (III) (this measurement is used in analysing cells outside of the ATMP fields). Furthermore, the data formatting adheres to standard conventions, and real‐time capability is specified. The evaluation of real‐time capability is conducted by using the symbols: ‘‐’ to indicate the absence of real‐time capability, and ‘+’ to denote its presence.

Type of measurement	MR	No.	Method of measurement	Data format^*^	Real‐time (±)	Reference
Off‐line	I	1	Cell‐based assays (MTT, Alamar Blue)	analog	−	[52, 53, 54, 55, 56]
		2	Digital polymerase chain reaction (ddPCR)	tabular	−	[57, 58]
		3	(Auto) fluorescence imaging	image	−	[59, 60]
		4	Hemocytometry	analog	−	[61, 62]
		5	High‐performance liquid chromatography (HPLC)	tabular, mzML, CDF, AnIML	−	[63, 64, 65, 66]
		6	Immunofluorescent staining	image	−	[67, 68, 69]
		7	Microscopy (optical, electron, atomic force, etc.)	image, tabular	−	[70, 71, 72, 73, 74]
		8	Off‐line pH probes	tabular	−	[75]
		9	Osmometry	tabular	−	[76, 77]
		10	Quantitative polymerase chain reaction (qPCR)	tabular	−	[57, 78–80]
		11	Timer	analog	−	
		12	Western Blot	tabular	−	[81, 82, 83, 84]
	III	13	Digital lensless holography	tabular, image	−	[85, 86]
		14	Brillouin microscopy	tabular, image	−	[87, 88, 89]
		15	Cytokine bead array (CBA)	tabular, FCS	−	[90, 91, 92]
		16	Gravimetric method	analog	−	[93, 94]
		17	Karyotyping	tabular, image, video	−	[95, 96]
		18	Liquid chromatography and mass spectrometry (LC‐MS)	tabular, mzML	−	[97, 98]
		19	Next‐generation sequencing (NGS)	FASTA	−	[99]
		20	Time‐lapse microscopy	tabular, image, video	−	[100, 101, 102, 103]
		21	Whole‐genome sequencing (WGS)	Fastq, BAM, BCL	−	[99, 104, 105]
At‐line	I	22	Enzyme‐linked immunosorbent assay (ELISA)	tabular	−	[106, 107]
		23	Endotoxin test	tabular, binary	−	[108, 109, 110]
		24	Flow cytometry	tabular, binary, FCS	−	[111, 112, 113, 114]
		25	Fourier transform infrared spectroscopy (FTIR)	tabular	−	[77, 115–118]
		26	Image analysis: Bright‐field / phase‐contrast microscopy/ fluorescence microscopy	image	−	[119, 120, 121, 122, 123, 124]
		27	Impedance‐based sensing	tabular, binary	−	[125, 126, 127, 128, 129, 130]
		28	Raman spectroscopy	tabular, ASCII	−	[75, 131, 132]
	II	29	Sterility testing (bioburden)	tabular	−	[133, 134]
		30	Environmental monitoring (optical, filtration, laser particle counters)	tabular	−	[135]
		31	Hyperspectral imaging	tabular, image	−	[136, 137]
		32	Osmometer	tabular	−	[77]
	III	33	Laser force cytology (LFC)	tabular	−	[138, 139]
		34	Automated hematology analysers (AHA)	tabular	−	[140, 141, 142]
		35	Bioluminescence imaging	image	−	[57, 129]
		36	Fiber optic sensors (pH, O_2_)	tabular	−	[143, 144]
		37	Near‐infrared (NIR) spectroscopy	tabular, JCAMP‐DX	−	[145, 146, 147, 148, 149]
		38	Spectrophotometry (UV‐Vis)	tabular	−	[150, 151, 152, 153]
On‐line	I	39	Timer integrated in others systems	tabular	+	
	II	40	Biosensors (Optical)	tabular	+	[154, 155, 156]
		41	Glucose and lactate sensors	tabular	+	[157, 158, 159]
		42	Resistance temperature detectors	tabular	+	[144, 155, 160, 161]
	III	43	pH meters	tabular	+	[162, 163, 164]
		44	Polarographic electrode	tabular	+	[143, 165, 166]
		45	Flow meters (ultrasonic, electromagnetic, Coriolis, thermal)	analog	+	[167, 168, 169]
In‐line	II	46	Infrared (IR) thermometers	tabular	+	[170]
	III	47	Thermal imaging cameras	tabular, image, video	+	[171, 172, 173]

*Note*: *Data formats:.

tabular: TXT files, CSV

binary: data using 0 or 1, typically digitalized analog signals

analog: not directly read by a machine, e.g., hand written data.

image: JPEG, TIFF, PNG.

video: AVI, MP4.

ASCII: used to encode alphabet characters and other symbols.

BAM: Binary Alignment Map.

JCAMP‐DX: used to store and exchange spectral and chromatographic data.

mzML /mzXML: XML (eXtensible markup language) based common file format for proteomics mass spectrometric data.

Fastq, fasta: text‐based representation for nucleotide sequences and amino acid (protein) sequences.

VCF: variant cell format.

CDF: common data format.

AnIML: analytical information markup language as standard for analytical chemistry and biological data.

BCL: base cell files.

FCS: flow cytometry standard.

CRAM: compressed alignment map.

In this manuscript, we distinguish between off‐line and at‐line measurement methods primarily based on the proximity and immediacy of sample analysis relative to the production environment. Off‐line measurements involve collecting samples from the production process and subsequently analysing them in a separate laboratory environment, which often introduces significant delays and increases contamination risks (e.g., traditional flow cytometry, qPCR performed in external labs). For example, a sample collected from the bioreactor is transferred to a separate laboratory room or building to measure cell viability using flow cytometry. At‐line measurements, in contrast, are performed close to the production site. Samples are manually collected but analysed immediately near the production equipment, minimizing transport time and reducing delays in obtaining results (e.g., automated cell counters placed next to bioreactors, rapid endotoxin tests performed on‐site). E.g., a sample is taken from the bioreactor and directly measured using a rapid, automated viability analyser placed adjacent to the bioreactor.

To illustrate practical differences among off‐line, at‐line, on‐line, and in‐line monitoring methods, commonly used examples from CAR T cell manufacturing include flow cytometry performed externally (off‐line) [111, 112, 114], rapid automated cell counters located adjacent to bioreactors (at‐line) [61, 62], automated glucose and lactate sensors integrated via bypass (on‐line) [157, 159], and integrated pH or dissolved oxygen sensors continuously measuring within bioreactors without sample removal (in‐line) [174].

Table 2 provides an overview of the five primary steps involved in CAR T cell production, along with the key parameters that need to be monitored to ensure successful completion of each step. The table also references viable measurement methods for each step, as detailed in Table 1. The overarching goal is to facilitate on‐line measurement during production by implementing a morphological box‐style measurement approach that will assist in identifying the most advantageous option. The term “morphological box‐style” denotes a systematic approach used in decision‐making and problem‐solving processes. Parameters are further categorized into core and research parameters. Core metrics include parameters that are already monitored within the production process and are recognized as essential for its proper and consistent operation. Their continuous monitoring is critical to ensure the proper functioning of the production process. Research metrics, on the other hand, are parameters that have been identified, tested, and measured during research activities. While they are not critical to the basic production process, they can provide additional insight or improvements in process monitoring. For example, metrics such as cell count and cell viability are typically measured and are integral to the process, so they are classified as core parameters. Parameters such as cell phenotype are categorized as both core and research, as the exploration of new phenotypes/parameters is ongoing [175].

**TABLE 2:** Main process steps of CAR T cell manufacturing with key parameters and measurement methods referring to Table 1.

CAR T manufacturing step	Core or research metrics	Parameter	Measurement method (see Table 1)
Selection	Core	Cell concentration	4, 7, 13, 23, 25, 26, 37
		Cell count	4, 7, 13, 23, 25, 26, 37
		Cell viability	4, 7, 13, 34, 25, 26, 27
		Processing time	11, **39**
		pH level	8, **42**
		Sterility	4, 10, 11, 21, 23, 26, 27, 37
		Temperature	35, **45, 46**
		Volume of blood processed*	4, 5, 18, 23, 25, 26, 32
		Cell activity state*	3
Activation	Core	Cell concentration	4, 7, 13, 23, 25, 26, 37
		Cell count	4, 7, 13, 23, 25, 26, 37
		Cell viability	4, 7, 14, 23, 25, 26, 27
		pH level	8, **42**
		Processing Time	11, **39**
		Sterility	4, 10, 11, 21, 23, 26, 27, 37
		Temperature	35, **45, 46**
		Cell heterogeneity*	3
	Research	Cytokine production*	6, 26, 27
		Proliferation capacity and upregulation of activation markers*	5, 12, 18, 21
		Stiffness*	7
Gene Delivery		CAR expression*	3, 7, 10, 12, 21, 23, 25, 34
		Cell concentration	4, 7, 13, 23, 25, 26, 37
		Cell count	4, 7, 13, 23, 25, 26, 37
		Cell identity*	3, 10, 23
		Cell morphology*	7, 13, 14
		Cell viability	4, 7, 13, 23, 25, 26, 27
		pH level	8, **42**
		Processing Time	11, **39**
		Sterility	4, 10, 11, 21, 23, 26, 27, 37
		Temperature	35, **45, 46**
		Vector copy number (VCN)*	2, 10
Expansion	Core	Cell concentration	4, 7, 13, 23, 25, 26, 37
		Cell count	4, 7, 13, 23, 25, 26, 37
		Cell morphology*	7, 13, 14, 23
		Cell phenotype*	2, 4, 23, 25, 26, 32, 37, 38
		Cell viability	4, 7, 13, 23, 25, 26, 27
		Glucose and lactate levels*	5, 21, 36, 38
		pH level	8, **42**
		Processing time	11, **3** **9**
		Sterility	4, 10, 11, 21, 23, 26, 27, 37
		Shaking speed (in bioreactor)*	integrated in bioreactor
		Temperature	35, **45, 46**
	Research	Cell phenotype*	2, 4, 23, 25, 26, 32, 37, 38
		Cytokine production*	21, 23
		Dissolved oxygen*	35, **43**
		Osmolality of the medium*	31
Formulation	Core	Cell concentration	4, 7, 13, 23, 25, 26, 37
		Cell count	4, 7, 13, 23, 25, 26, 37
		Cell phenotype*	2, 4, 23, 25, 26, 32, 37, 38
		Cell viability	4, 7, 13, 23, 25, 26, 27
		Genetic stability*	4, 10, 17
		pH level	8, **42**
		Processing Time	11, **39**
		Sterility	4, 10, 11, 21, 23, 25, 26, 37
		Temperature	35, **45, 46**

*Note*: Available automated on‐line measurements are marked in bold and at‐line measurements that can be automated are underlined. Core metrics are essential parameters routinely monitored for proper production function. Research metrics, identified during research, offer additional insights for process improvement. Some parameters (e.g., cell count, viability, sterility, temperature, pH, and processing time) are critical throughout the entire CAR T cell manufacturing process and therefore appear across multiple steps. In contrast, step‐specific parameters that characterize unique aspects of individual process stages are marked with an asterisk (*x*) in the table (e.g., CAR expression during gene transfer, or shaking speed in expansion).

In pursuit of this goal, it is imperative that the production process be broken down into discrete steps, using a modular production approach. This approach ensures that each step in the process is performed accurately, allowing subsequent steps to be performed in separate locations, decoupled from the previous steps.

Multiple measurement options are available for most parameters. Flow cytometry (No. 24), enzyme‐linked immunosorbent assay (ELISA) (No. 22), spectroscopy (Raman, NIR) (No. 28 and 37), hemocytometry (No. 4), and impedance‐based sensing (No. 27) are among the most widely used methods. However, other measurement methods have already shown promising results in studying cell cultures outside CAR T cell production, such as hyperspectral imaging (No. 31) [176].

Table 2 shows that the important key parameters, cell viability and cell number, from which many other parameters such as cell concentration can be calculated, can currently only be measured off‐line. To achieve continuous monitoring of these parameters in real time, the available measurement methods for on‐line or in‐line measurement need to be automated. Although many automated systems for these measurements on the market are called on‐line measurement solutions, they are categorized here as at‐line solutions based on the classification presented in Figure 2. Currently available integrated bioreactor devices predominantly feature rudimentary quality assessments encompassing gas concentration, temperature, and pH. Molecular and cellular quality metrics, including cell viability, cell count, cellular identity, purity, and CAR receptor expression, necessitate evaluations using samples that are manually procured and processed [2]. Thus, we have decided on this categorization primarily because a sample still needs to be manually taken from the production batch and tested, even with these automated systems.

Another approach to continuously monitoring these parameters in real time is the development of alternative measurement technologies or methods. One example is optical biosensors. Optical biosensors, which utilize light to detect and analyse biochemical interactions, represent a significant progression in biotechnological tools due to their high sensitivity and the ability to produce real‐time label‐free results [177]. Their primary mechanism of action is the evanescent wave technique, which detects small changes in the behavior of light as it interacts with a biological sample. The two most common types of biosensors are surface plasmon resonance (SPR) biosensors and waveguide‐based biosensors. SPR biosensors employ a thin layer of gold, which is affected by biological interactions, leading to real‐time observations of the modulated optical behavior. Waveguide‐based sensors direct light through a specific material and detect light wave interferences that provide information about biological interactions.

The recently increasing interest in biosensors is mainly due to their ability to detect various substances, including molecules and pathogens [178, 179, 180]. Applying optical label‐free platforms in live cell biology remains relatively unexplored [181, 182, 183].

Providing real‐time data on the interaction between immune cells and tumors is essential. Sensors can detect proteins and other substances that affect tumor progression and the immune response in real time because both cancer cells and immune T cells produce substances that modulate tumor progression and the immune response [181]. This facilitates an understanding of both the mechanisms of immune cell function and the dynamics of tumor response. For example, optical biosensors have been used in academic research to monitor the secretion dynamics of the vascular endothelial growth factor (VEGF), a protein fundamental to the formation of blood vessels and tumor progression [184]. Using these sensors, researchers have obtained real‐time insights into the secretion patterns of VEGF from live cancer cells. As a result, they have enhanced our understanding of tumor trajectories and responses to different stimuli.

Another example of an alternative measuring approach is the use of soft sensors [185], which are already well established in traditional bioprocessing applications [186]. Recently, their use has also been explored in the context of CAR T cell manufacturing, for example, in the prediction of viable cell density based on process variables [187]. Soft sensors combine software and sensors and are computational algorithms or models that estimate process variables or quality parameters using real‐time process data. They are implemented in software and can provide cost‐effective and efficient solutions for monitoring, controlling, and optimizing industrial processes [188, 189]. Soft sensors operating in real time are used for a wide range of applications in process industries, including bioprocessing, chemical processing, and energy production [189]. However, the lack of adequate process models for many biological processes acts as a hindrance to the implementation of soft sensors in biological production [190]. Biological processes are often complex and make predicting outcomes challenging. This raises the issue of choosing between model‐based and data‐driven approaches. Model‐based methods demand a sound comprehension of the underlying biological mechanisms but may be incomplete or inaccurate due to the complexity of biological processes [187]. Data‐driven approaches leverage large amounts of data to identify patterns and relationships but can be susceptible to noise and uncertainty. A promising solution may be found in hybrid models that integrate both model‐based and data‐driven approaches [191, 192, 193, 194]. Such models can fuse existing biological knowledge and the benefits of extensive datasets for more precise predictions and controls [192]. By implementing hybrid models, the difficulties of developing soft sensors for CAR T cell production can be surmounted, and process monitoring and control can be significantly improved and made more reliable.

### 4.3 Monitoring Genomic Stability and Risk of Secondary Malignancies

A critical safety concern in the production and application of CAR T‐cell therapies is the potential development of secondary primary malignancies (SPMs). These malignancies may arise due to the genetic manipulation inherent in CAR T‐cell manufacturing processes, posing long‐term risks to patient safety. Although rare, such cases have been reported—for example, the development of CAR‐positive T‐cell lymphomas or therapy‐related myeloid neoplasms following CD19‐directed CAR T‐cell therapy [195, 196]. These findings underscore the importance of long‐term monitoring strategies and careful assessment of genomic integrity during and after manufacturing.

A systematic review and meta‐analysis of 5,517 patients receiving CAR T‐cell therapy for lymphomas and myelomas reported 326 cases of SPM within a median follow‐up period of 21.7 months, with hematologic malignancies being the predominant type [196]. The genetic modifications introduced during CAR T‐cell production, particularly those involving viral vector‐mediated gene transfer, can increase the likelihood of insertional mutagenesis and consequent genomic instability. Such genomic alterations could potentially predispose treated patients to secondary malignancies [195].

To mitigate these risks, robust monitoring strategies must be employed. Existing methods such as digital PCR (ddPCR), next‐generation sequencing (NGS), and karyotyping provide valuable tools for assessing genomic stability throughout the manufacturing process [95, 197, 198]. ddPCR, in particular, can quantify vector copy numbers (VCN), enabling precise monitoring of genomic insertions. NGS offers comprehensive genomic analysis capabilities, allowing the detection of off‐target genomic modifications and mutations. Karyotyping remains valuable for identifying gross chromosomal alterations, providing critical insights into genomic integrity.

However, these techniques are predominantly performed off‐line, limiting their real‐time application in manufacturing settings.

## 5 Limitations of Research and Current Systems

### 5.1 Limitations of this Review

This review aimed to investigate existing solutions for monitoring process parameters in CAR T cell production and to identify parameters of particular importance for maintaining process control and ensuring product quality. The approach was based on a systematic analysis of scientific publications and review articles available through peer‐reviewed journals and conference proceedings. However, this method also introduces several limitations that should be acknowledged to contextualize the findings presented.

Firstly, the review does not encompass a direct comparison between established measurement methods and monitoring functionalities within current commercial manufacturing platforms [199, 200]. While these all‐in‐one devices represent widely used solutions in industrial (and clinical) CAR T cell production, their proprietary designs and limited publicly available data constrain the ability to benchmark literature findings against current industrial practice. Future work could benefit from a device‐centered analysis or direct access to non‐public data.

Secondly, limited data availability for certain process parameters—particularly those labeled as research metrics labeled in Table 2—presented a challenge. These parameters are often not fully disclosed in the literature due to confidentiality or intellectual property protection. In this context, patent analysis may provide a useful supplement to scientific publications and could help to bridge knowledge gaps regarding upcoming technological innovations and developmental trends.

Another constraint relates to the relevance of laboratory‐scale findings for large‐scale production. Measurement approaches and parameters that are feasible in academic research may not be applicable or scalable to GMP‐compliant manufacturing processes. Therefore, further work is needed to assess which parameters truly influence product quality and consistency under real‐world conditions.

Particularly in decentralized or modular production models, key parameters that influence the next manufacturing step must be incorporated in the system, too. Parameters such as cell density and phenotype measured during expansion should be forwarded to the formulation step, where they directly influence formulation decisions. Conversely, for parameters like pH or osmolality that are relevant only locally, a certificate confirming they were within specification may be sufficient. This approach could streamline data handling and support traceable, compliant manufacturing.

Finally, while this review emphasizes monitoring technologies, it does not assess how monitoring data is currently integrated or used in decision‐making in CAR T cell production. This is particularly relevant when considering the goal of establishing adaptive and automated manufacturing strategies. A deeper understanding of data flow, standardization, and usage would enhance efforts to develop digital twins and predictive control strategies.

### 5.2 Limitations of Existing Monitoring Systems

Although significant progress has been made in monitoring systems for CAR T cell manufacturing, several critical limitations persist. Most currently available monitoring technologies are predominantly offline or atline methods, meaning samples must be manually collected and processed separately, introducing potential contamination risks and delays in obtaining results. Such delays limit real‐time decision‐making capabilities and can impact the timely detection of critical deviations.

#### 5.2.1 Selection and Apheresis: Dealing With Variability at the Source

Monitoring challenges begin with the initial collection of patient cells. Inter‐patient variability can significantly impact downstream manufacturing outcomes [201]. Factors such as the patient's disease state, prior treatments, and age influence T cell functionality and phenotypes. Monitoring cell count, viability, and phenotypic markers (e.g., CD3, CD4, CD8 ratios) at the apheresis stage is crucial to assess starting material quality [202]. However, many platforms lack rapid, decentralized testing capabilities at the clinic, leading to delays or missed opportunities to adjust process parameters early. Real‐time feedback mechanisms could improve the selection and acceptance criteria for incoming cellular material.

#### 5.2.2 Activation and Transduction: Real‐Time Insights Into Early Process Shifts

In the activation and gene transfer phase, key indicators such as CD69, CD25, and CD137 expression levels provide information about T cell responsiveness and activation kinetics [203]. However, monitoring of these markers is often limited to off‐line flow cytometry. Real‐time monitoring of transduction efficiency—via CAR expression or VCN—is also constrained by a lack of in‐line methods. Measurements are typically done post‐transduction and off‐line using PCR or antibody staining, which delays feedback and reduces the ability to adapt transduction protocols in real time [57, 58].

The development of in‐line or on‐line tools to monitor early gene expression or reporter activity could provide valuable process insights and enable immediate adjustments. Additionally, integrating feedback from activation monitoring into the design of vector dosing strategies could enhance both efficiency and consistency.

#### 5.2.3 Expansion: Continuous Control of Growth and Metabolism

Expansion is the most time‐intensive step and highly sensitive to environmental conditions. Monitoring nutrient consumption (e.g., glucose, glutamine) and waste accumulation (e.g., lactate, ammonia) can inform feeding strategies and detect suboptimal culture conditions [204]. Morphological changes, cell size distribution, and impedance are potential early indicators of culture stress or failure, but are rarely monitored in‐line [205].

Sensors for pH, dissolved oxygen, and temperature are standard in conventional stirred‐tank bioreactors and well established in CHO‐ and HEK‐based processes [174]. However, their use and validation in the context of primary human T cell cultivation—especially in GMP settings—remains limited and requires further adaptation.The integration of dielectric spectroscopy and multi‐angle light scattering technologies into single‐use bioreactor systems offers a promising avenue for real‐time tracking of viable biomass and cell aggregation states [174]. However, their validation in clinical manufacturing contexts remains pending. Furthermore, strategies to combine metabolic and morphological data into soft sensor frameworks may enhance process control.

#### 5.2.4 Formulation and Final Control: Potency, Stability, and Release Readiness

During formulation, cells are washed, concentrated, and suspended in the final product medium. Critical measurements at this stage include viability, phenotype confirmation, and potency testing (e.g., cytokine production, cytotoxicity assays). However, many of these tests are labor‐intensive and performed off‐line under release testing protocols. There is a lack of in‐line or rapid potency assays that could allow early detection of compromised products before batch finalization [206]. Additionally, cold chain monitoring during final packaging is essential for maintaining product integrity but is often treated as a separate logistical task rather than a component of integrated monitoring.

Emerging rapid potency platforms based on surface marker activation profiles or metabolic readouts could reduce reliance on long assay times and streamline batch release [207]. Moreover, embedding temperature and vibration sensors directly into shipping containers may support traceable and secure cold chain management.

#### 5.2.5 Cross‐Cutting Challenges: Data Integration, Automation, and Regulatory Alignment

Several overarching limitations span the entire manufacturing process. Soft sensors and hybrid models, which combine limited measured data with predictive algorithms, are promising but underdeveloped in CAR T manufacturing due to the complexity of biological systems and lack of robust training datasets [185, 186].

Digital twins—virtual representations of the production process—could enable simulation and optimization, but their implementation is hindered by a lack of standardization in data formats and data sharing between systems and manufacturers [208]. Similarly, AI/ML applications are beginning to support quality predictions but face validation challenges under GMP regulations [209].

From a regulatory perspective, the lack of harmonized standards for measurement, data reporting, and equipment qualification slows adoption. While ICH guidelines (e.g., Q8–Q11) provide general frameworks, specific regulatory expectations for in‐line monitoring in ATMP production are still evolving. Ensuring traceability, cybersecurity, and compliance in digital systems is a growing priority, particularly in decentralized or modular manufacturing setups [182, 183].

Collaborative initiatives between industry, regulatory bodies, and standardization organizations are needed to develop validation frameworks for emerging monitoring technologies. The establishment of common data models and qualification protocols would significantly enhance the adoption and comparability of digital systems in cell and gene therapy manufacturing.

## 6 Conclusion and Outlook

To make CAR T cell therapy accessible to more patients, some aspects of production need to be improved by new biological technologies or by improving automation and digitalization. Current monitoring technologies used in CAR T cell production make it challenging to implement digital twins that require accurate and comprehensive data collection. With new monitoring technologies, the implementation of digital twins could be enabled, representing a significant step towards more modular and flexible CAR T cell production processes. This modularity will allow for the decoupling of individual steps of the production process, ultimately dissolving the traditional all‐in‐one production line and enabling a more efficient and adaptable production system.

This work aimed to identify the key parameters to be monitored during CAR T cell production. These parameters and their different measurement methods were derived from the literature review. In our view, strict off‐line measurement methods are bottlenecks hindering the realization of digital monitoring. These bottlenecks need to be overcome to enable continuous and automatic real‐time measurement. Each key step of the CAR T cell production process was mapped to possible measurement methodologies.

While developing a potential concept for monitoring CAR T cell production is a positive step towards more modular and flexible production processes, implementing and evaluating this concept in concrete applications will be necessary in future work. Further research and adaptations to measurement methodologies and data structures will be required to optimize the effectiveness of this approach for different variants of CAR T cell production. The next step is to select the measurement methods as proposed here to realize the most continuous on‐line measurement possible for the CAR T cell production. Furthermore, research should focus on describing standards for either production or communication or for both. Decentralized, intelligent on‐site production in line with biointelligent principles can provide immediate support to more patients. It is only possible through fully digitalized, automated, and modular production. Additionally, the development of an information modelling framework for CAR T cell production has to be addressed. Such a framework should include critical aspects such as data organization, storage and anonymization. With respect to modular production, a novel approach is needed to effectively manage the different responsibilities of each step in the process by creating a modular data model. In particular, both automated and manual production processes are still unexplored and need to be considered. The creation of interfaces that allow adaptive reconfiguration of the system is an area that needs further investigation. The aim is to create a versatile framework model that can map the different production steps and provide a comprehensive process perspective, as it is clear that the production of CAR T cells is characterized by its non‐standardized nature. Therefore, flexible implementation approaches are essential. In order to advance the optimization of monitoring and measurement methods at the production level, future research should focus on this aspect.

Accurate words and terms should be used in the subject‐specific vocabulary when they convey the meaning more precisely than a similar non‐technical term. Research in this field may aid in developing distinct guidelines for selecting and adjusting measurement parameters in CAR T cell manufacturing, thus overcoming the disparity between the laboratory and production.

Further attention must be paid to data security, especially data storage and anonymization. Possible solutions such as cloud‐based resources, specialized systems, and data lakehouses still need to be thoroughly explored. The fundamental importance of anonymizing data to protect patient privacy and maintain ethical standards in CAR T cell therapy needs to be fully explored to ensure data integrity and data applications.

## Author Contributions

**Arber Shoshi**: conceptualization, methodology, formal analysis, writing – original draft preparation, writing – review and editing. **Yuchen Xia**: conceptualization, formal analysis, investigation, writing – original draft preparation. **Andrea Fieschi**: conceptualization, formal analysis, investigation, writing – original draft preparation. **Yannick Baumgarten**: writing – review and editing. **Andrea Gaißler**: writing – review and editing. **Thomas Ackermann**: writing – review and editing, supervision, project administration. **Peter Reimann**: writing review and editing, supervision, project administration. **Bernhard Mitschang**: writing review and editing. **Michael Weyrich**: writing review and editing. **Thomas Bauernhansl**: writing – review and editing. **Robert Miehe**: writing review and editing, supervision. All authors have read and agreed to the published version of the manuscript.

## Conflicts of Interest

The funders had no role in the design of the study; in the collection, analyses, or interpretation of data; in the writing of the manuscript; or in the decision to publish the results.

## Data Availability

Data sharing not applicable to this article as no datasets were generated or analysed during the current study.
